# Antibacterial Polysaccharides in Dental Implantology

**DOI:** 10.3390/md23080321

**Published:** 2025-08-04

**Authors:** Lubica Hallmann, Mark Daniel Gerngroß

**Affiliations:** 1 School of Dentistry, Kiel University, 24105 Kiel, Germany; 2 Institute of Material Science, Faculty of Engineering, Kiel University, 24143 Kiel, Germany; mdg@tf.uni-kiel.de

**Keywords:** implant infections, antibiotic resistance of bacteria, antibacterial marine polysaccharides, plant polysaccharides, biocompatibility, bioactivity

## Abstract

**Background:** The aim of this review is to summarize and evaluate the properties of antibacterial polysaccharides for application in dental implantology to identify knowledge gaps and provide new research ideas. **Methods**: The electronic databases PubMed, Medline, ProQuest, and Google Scholar were used to search for peer-reviewed scientific publications published between 2018 and 2025 that provide insights to answer research questions on the role of antibacterial polysaccharides in combating pathogens in dental implantology without triggering immune reactions and inflammation. Further research questions relate to the efficacy against various dental pathogens and the understanding of the antibacterial mechanism, which may enable the development of functionalized polysaccharides with long-term antibacterial activity. **Results:** Biomedical implants have revolutionized medicine but also increased the risk of infections. Implant infections are a major problem in implantology and lead to implant failure and replacement. An antibacterial coating could be an excellent strategy to extend the lifespan of implants and improve the quality of the patient’s life. Bacterial resistance to antibiotics poses significant challenges for researchers, forcing them to search for new ways to prevent bacterial infections in implantology. Antibacterial natural polymers have recently received considerable research attention due to their long-term antibacterial activity. Polysaccharides from marine sources, such as chitosan and alginate, or pectin, xanthan, etc., from various plants, appear to be promising biopolymers for such applications in implantology due to their antibacterial activity, biocompatibility, and osteogenic properties. The antibacterial activity of these natural biopolymers depends on their chemical and physical properties. Nanopolysaccharides exhibit higher antibacterial activity than conventional polysaccharides, but their toxicity to human cells must be considered. Their antibacterial activity is based on the disruption of bacterial DNA or RNA synthesis, increased cell wall permeability, membrane disruption, and cytoplasmic leakage. **Conclusions:** Polysaccharides are a class of natural polymers with a broad spectrum of biological activities. They exhibit antioxidant, immunomodulatory, anticoagulant, anticancer, anti-inflammatory, antibacterial, and antiviral activity. Furthermore, polysaccharides are non-cytotoxic and exhibit good biocompatibility with osteogenic cells. Bactericidal polysaccharides are attractive new antibacterial materials against implant infections and open up new perspectives in implantology.

## 1. Introduction

Dental and orthopedic implants have revolutionized the surgical and restorative aspects of dentistry and orthopedics, as they offer an excellent alternative for repairing damaged bones or missing teeth [1,2,3,4]. However, during the surgical procedure, bacteria can adhere to the implant surface and form a biofilm that triggers peri-implantitis [5]. The durability of an implant depends on many factors, such as mechanical properties, surface roughness, implant abutment, connection design, implant geometry, implant position, bone density, surface materials, micro-gaps, corrosion resistance, biocompatibility, osseointegration, and antibacterial activity of the materials [6,7,8,9,10,11,12]. All of these factors influence the integration of the implant into the bone tissue and the mechanical stress distribution at the bone–implant interface [9].

**Figure 1:**
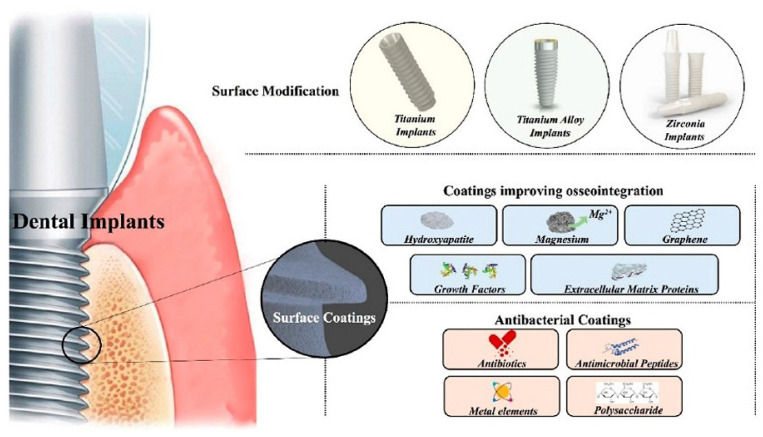
A schematic illustration of surface modifications and functional coating of dental implants [14].

Titanium (Ti-ß) and Ti-6Al-4V are used for dental implants. The formation of a stable and dense oxide layer on the surface of these materials is the reason for their good biocompatibility and corrosion resistance. However, damage to this oxide layer by biocorrosion caused by pH, debris, bacteria, or abrasion can lead to the release of metal ions, which can trigger inflammatory reactions and thus implant failure [13]. However, recent research approaches are moving towards avoiding the use of potentially tissue-damaging elements such as vanadium in titanium alloys [6]. To avoid these problems associated with titanium alloys, zirconia is used as a dental material for implants because it has superior aesthetic properties compared to titanium and has excellent properties, such as low elastic modulus, low thermal conductivity, and high biocompatibility. However, the lack of bioactivity and antibacterial activity compromises the long-term stability of zirconia dental implants. Modifications of the zirconia implant surface are necessary to improve cell adhesion, proliferation, and differentiation, as well as its antibacterial activity (Figure 1) [14].

Surface topography is a very important aspect in the design and manufacturing of dental implants, as it directly influences bioactivity, osseointegration, and bacterial infections. Infections at the implant site can hinder or even completely prevent osseointegration, potentially leading to implant failure or the need for surgical removal [15].

**Figure 2:**
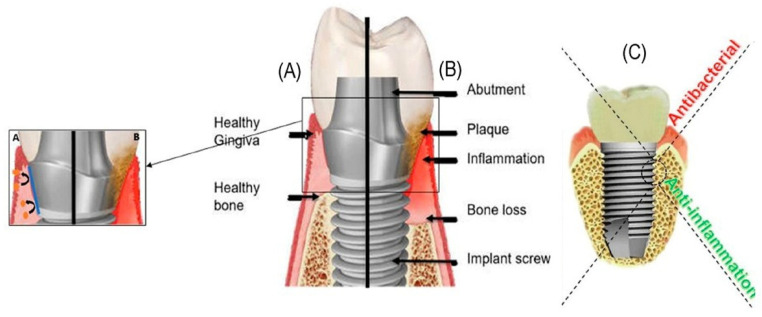
Biofilm formation process around a dental implant and the dental structure. (**A**) Implant coating preventing bacterial colonization. (**B**) Dental plaque formation [8]. (**C**) Antibacterial and anti-inflammatory coating of a dental implant [14].

Peri-implant diseases such as peri-implant mucositis and peri-implantitis are biofilm-related inflammations of the peri-implant tissue. Peri-implant mucositis is considered a precursor to peri-implantitis, a disease that can progress rapidly and lead to advanced bone loss and eventual implant loss and removal. Early detection of peri-implant diseases and early intervention are crucial for implant longevity, as well as patient satisfaction and quality of life [13,16]. Figure 2 shows the difference between healthy (A) and inflamed gingiva (B), as well as the influence of peri-implantitis on bone loss.

Figure 2C schematically shows the different areas of action of the antibacterial and anti-inflammatory coating on the implant surface. Bone trauma that occurs during implantation creates a fibronectin-rich blood clot pillar, allowing cells to form new tissue there. Osteogenic cells begin to release mineralized collagen between the implant and the host, leading to the formation of new bone tissue. This freshly formed bone tissue prevents implant movement and thus increases the survival rate of the implant [14]. Bacterial infections and inflammatory reactions can significantly impair the process described above. Therefore, it is important to coat the implant surface with antibacterial and anti-inflammatory layers.

**Figure 3:**
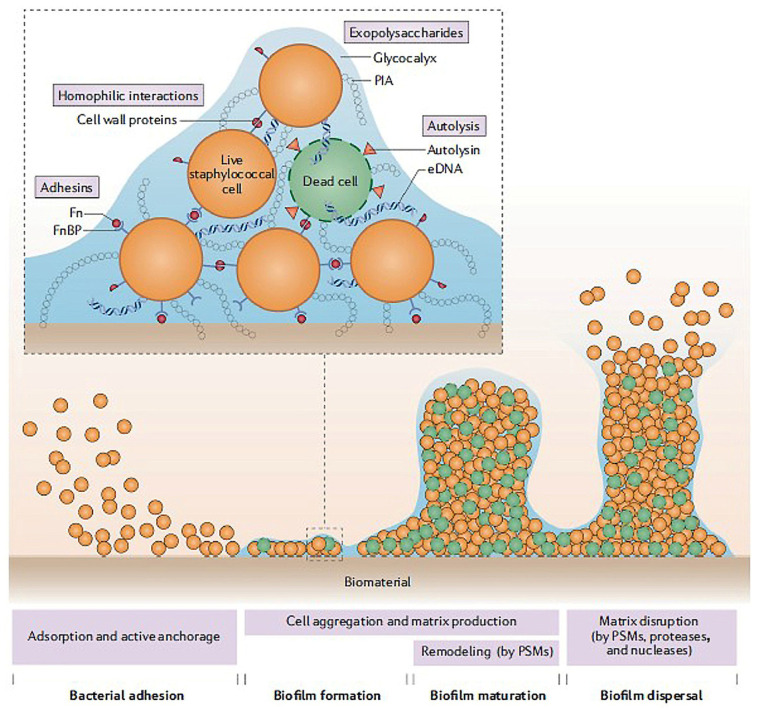
Stages of a staphylococcal biofilm formation. Stable anchorage of bacteria is generally followed by the formation of a biofilm. Intercellular interactions mediated by adhesins and cell wall proteins lead bacteria to cluster together, forming microcolonies. For example, in *S. aureus*, fibronectin-binding proteins (FnBPs) bind to fibronectin (Fn) molecules, forming a bridge. This interaction promotes bacterial aggregation. The production of extracellular polymeric substances is part of the biofilm maturation process, in which the biofilm matrix progressively builds up and larger bacterial aggregates, called towers, develop. In *S. aureus* and *S. epidermidis*, the mechanisms of biofilm formation include the expression of polysaccharide intercellular adhesin (PIA) and the release of extracellular DNA (eDNA) derived from bacterial autolysis and from dead host cells. In *S. epidermidis*, the β-subclass of phenol-soluble modulins (PSMs) contributes to biofilm structuring and leads to the formation of the characteristic water channels 95, which are observed in mature biofilms. In *S. aureus* and *S. epidermidis*, PSMs are also involved in biofilm dispersal, together with proteases and nucleases [22]. Reprinted with the permission of SpringerNature.

Biofilm formation plays a crucial role in the pathogenesis of implant-associated infections [15,16,17,18,19,20]. Biofilms are structurally complex and should be considered as a dynamic system that can protect bacteria from host defense mechanisms and antibiotics [20,21]. The three-dimensional structure of the bacterial biofilm can act as a natural barrier against antibiotics and reduce the biofilm’s susceptibility to antibiotics (Figure 3) [22].

**Figure 4:**
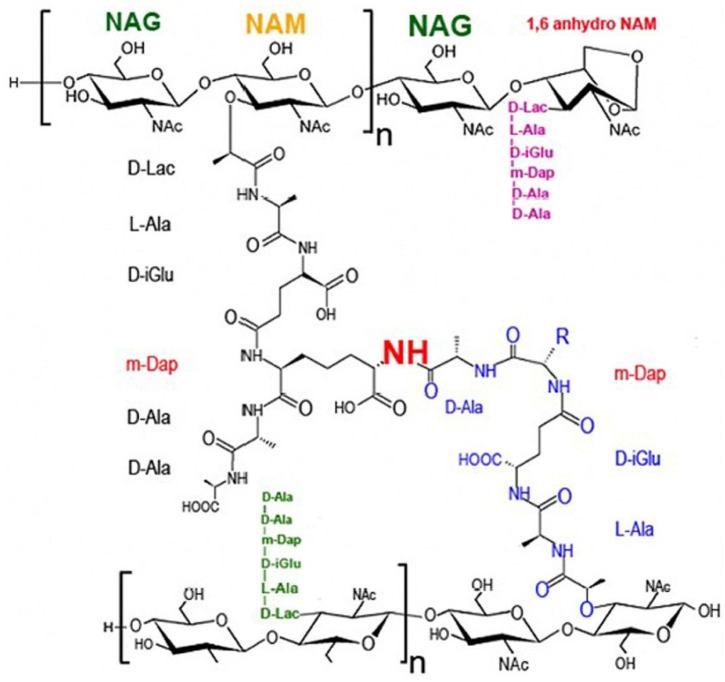
The bacterial cell wall backbone, peptidoglycan. Shown are the two glycan strands (in black). Peptide stems are depicted in black (left side), and the second peptide stem is depicted in blue. Note the cross-linking NH (in red) via the two unusual amino acids m-diaminopimelic acid (m-Dap, in red) and the presence of D-alanine in the peptide stems. Two more peptide stems (green and pink) are depicted, which can interact to build the next cross-linking between glycan strands [23]. Reprinted with the permission of ASM.

Bacteria are roughly divided into Gram-positive and Gram-negative based on the thickness of their cell walls (peptidoglycan). Gram-positive bacteria have thick cell walls (about 20–80 nm), while Gram-negative bacteria have thin cell walls (<10 nm). Bacterial cell walls provide vital structure for bacteria, protecting them from their often-hostile environment. Composed of unique components, they determine the shape of the bacteria, provide support for ligands and proteins to attach to host cells, offer receptors for drugs or viruses, represent the major targets for antibiotics, provide structures for immunological differentiation and variation, and can cause symptoms of disease in animals and humans. The main backbone of the bacterial cell wall is peptidoglycan, also called murein, which consists of repeating linear units of the disaccharide *N*-acetylglucosamine (NAG) linked to *N*-acetylmuramic acid (NAM). The disaccharides are cross-linked via often flexible pentapeptide amino acid chains, forming a mesh-like framework. Chemically, the peptidoglycan consists of alternating β-1,4-linked *N*-acetylglucosamine (GlcNAc;NAG) and *N*-acetylmuramic acid (MurNAc, NAM, a variant of GlcNAc with a D-lactate attached to the C-3 by an ether bond) (see Figure 4) [23].

**Figure 5:**
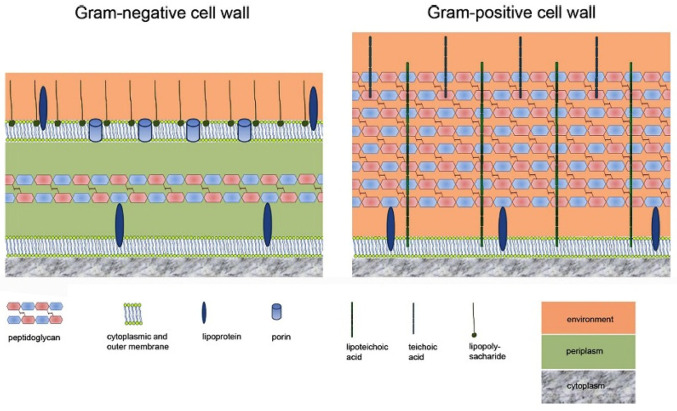
Schematic drawing of Gram-negative and Gram-positive cell walls. A characteristic of Gram-negative cell walls is the presence of two membranes: the cytoplasmic membrane and the outer membrane. Between these membranes is the periplasmic space, in which a very thin layer of peptidoglycan is found; lipopolysaccharides are attached to the outer membrane, and porins are inserted in the outer membrane. A thick layer of peptidoglycan and the lack of an outer membrane are the main characteristics of Gram-positive cell walls; instead of lipopolysaccharides, Gram-positive bacteria have lipoteichoic acid and teichoic acid localized in the cell wall [23]. Reprinted with the permission of ASM.

Although Gram-positive bacteria have thicker cell walls than Gram-negative bacteria, antibiotics can easily penetrate peptidoglycan. However, this is not possible with Gram-negative bacteria because they have an outer membrane that serves as a protective layer and is essential for survival (Figure 5) [23].

**Figure 6:**
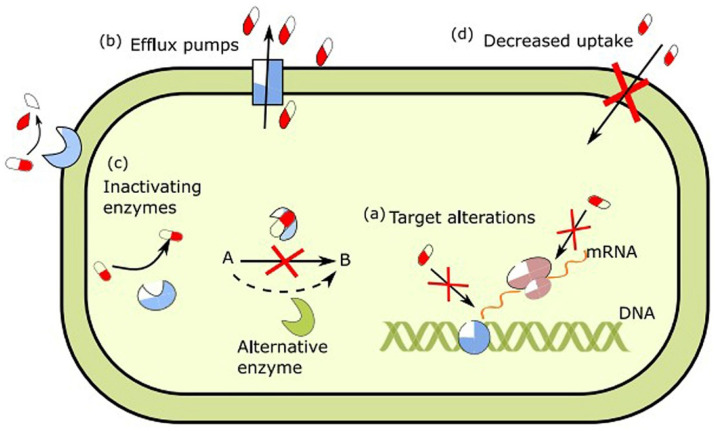
Antibiotic resistance mechanisms in bacteria occur in various ways. (a) Bacteria prevent the effectiveness of antibiotics by altering their targets, (b) by pumping antibiotics out of the cell through efflux pumps, (c) by producing enzymes that destroy antibiotics, and (d) and by impermeating the cell membrane, thus reducing drug uptake [24]. Reprinted with the permission of Elsevier.

There are three fundamental mechanisms of antimicrobial resistance: (a) enzymatic degradation of antibacterial drugs, (b) intracellular alterations and modifications of antibiotic targets, such as ribosomes and DNA proteins of bacteria, and (c) alterations in membrane permeability to antibiotics (Figure 6) [24].

Bacteria can evade both antibiotics and host defenses by hiding inside host cells. Up to 8% of *S. aureus* cells invade osteoblasts within 2 h. Inside these cells, *S. aureus* evades the intracellularly inactive antibiotics and activates professional phagocytes. Later, the bacteria can induce apoptosis of host cells and colonize the implant surfaces.

Several factors promote the bacterial colonization of implant surfaces. These include the surgical procedure itself, which provides a direct pathway for inflammation, the implant itself, which leads to metabolic exhaustion of neutrophils, making them less able to eliminate bacteria, and low blood vessel density around the implant, which prevents the timely arrival of immune cells and antibiotics [25,26,27,28,29,30,31].

In bacterial infections, pathogens typically utilize glycoconjugates covering mammalian cells to recognize and bind to host cells. The alteration of glycans reflects pathogens’ strategy of exploiting the host surface to evade its defense mechanisms [32,33,34,35,36,37,38].

Two important antibacterial strategies for modifying or altering implant surfaces are currently the focus of research: (a) preventing bacterial adhesion and bacterial accumulation or reducing the number of adherent bacteria and (b) killing bacteria that come into contact with the implant surface (reducing the viability of adherent bacteria) [39,40].

Numerous techniques have been developed to improve the antibacterial properties of implant surfaces. These can be roughly divided into the categories of surface modification and surface coating. Surface modifications include altering the surface free energy, hydrophobicity, and roughness [41,42,43,44,45,46,47,48,49,50,51,52,53]. For implant viability, it is crucial that the implant surface can prevent primary adhesion of microbial cells by either repelling or killing the invading bacteria. Both strategies delay or even prevent biofilm formation. However, they also have some disadvantages. Bacteria-resistant surfaces usually work well in the short term. In the mid- and long-term, contamination of such surfaces inevitably occurs with prolonged use, as the modified surface possesses no antibacterial effect and the bacteria can overcome the coating’s non-stick properties. Bacteria-killing surfaces are more effective at preventing the formation of bacterial biofilms due to their inherent bactericidal activity. The problem is that such surfaces can exert a toxic effect on human cells. The accumulation of dead bacteria on such a surface can not only reduce bactericidal activity but also trigger an immune response and cause inflammation [41].

Modifications of the implant surface resulting in accelerated osseointegration significantly contribute to mitigating biofilm formation and thus decrease the risk of peri-implantitis while achieving rapid loading of the damaged bone at the recipient site [54,55,56].

The immunological microenvironment can influence soft tissue integration and can be modulated by the surface properties of the implant. [54]. Immune cells can promote fibroblast formation, remove wound debris through phagocytosis, and produce enzymes that support soft tissue reorganization. Strong soft tissue adhesion is the first barrier against the invasion of bacterial pathogens. However, the risk of bacterial adhesion also increases with high permeability of the peri-implant soft tissue [54,55,56,57].

In the early stages of implant development, the focus was on the effect of microscale surface modification. More recently, the focus has shifted towards the nanoscale [56]. Different surface textures exhibit different effects on osseointegration and antimicrobial activity. Rough surfaces, such as those created by sandblasting, acid etching, or laser treatment, provide a highly increased surface area for the adhesion of bone, so that faster and stronger integration is promoted, increasing the external stability of the implant [58,59,60,61,62].

**Figure 7:**
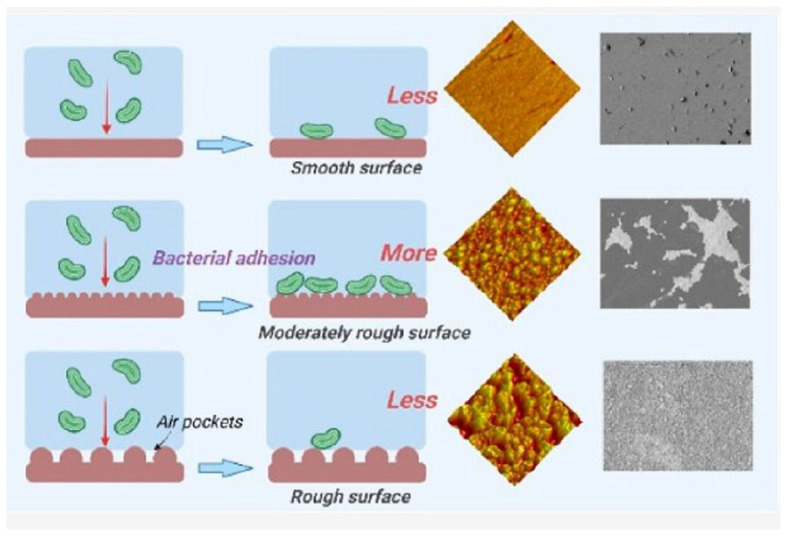
Influence of the roughness on the bacteria adhesion of the implant surfaces [47].

Surface properties that influence molecular interactions, cellular responses, and bone regeneration can significantly determine implantation success. Robust and stable soft tissue integration is required for the long-term function of dental implants. The long-term success of implants is influenced by their integration and the prevention of infections [63,64,65,66]. However, when designing an implant surface, a balance must be found between antimicrobial activity and the desired osteoconductive properties, as a rough implant surface can also promote biofilm formation in the long term [55]. Figure 7 illustrates the influence of surface roughness on bacterial adhesion. For hydrophobic surfaces, both a higher effective surface area with increasing roughness and reduced activation energy enhance bacterial adhesion [47,48,49].

For superhydrophobic surfaces, the reduction in bacterial adhesion decreases with increased surface roughness, which is due to the existing air gaps that strongly reduce the effective surface area, allowing for direct contact with the bacteria [47].

Coating implant surfaces is one of the most important methods for improving clinical efficacy. Antibacterial materials used to coat implants must meet certain requirements, such as biocompatibility and bioactivity, as their main function is to facilitate implant osseointegration [65]. Implant surfaces should allow the adhesion and growth of host cells while preventing bacterial adhesion and colonization. The balance between antimicrobial properties and biocompatibility is difficult to achieve. Typically, a surface that supports host cell adhesion is also beneficial for bacteria with similar adhesion mechanisms as host cells [37,38]. Antibacterial treatment of implant surfaces during the initial phase of biofilm formation is more effective than treating mature biofilm that is enveloped and protected by its own extracellular polymeric substances [8,22,67,68].

**Figure 8:**
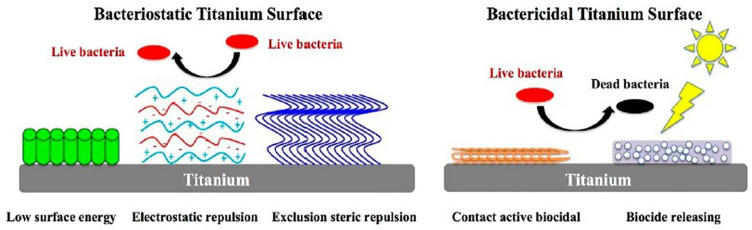
Various examples of antimicrobial surfaces according to the mechanism of action: bacteriostatic or bactericidal surfaces. The prevention of biofilm formation by an antibacterial coating is the best way to prevent primary adhesion or killing approaching bacteria [14].

To increase the biological activity of implant surfaces, various surface coating methods are currently being developed [69,70,71]. Figure 8 schematically presents different strategies for modified titanium implant surfaces with a functional coating to prevent bacterial adhesion and biofilm formation (bacteriostatic) or to kill bacteria (bactericidal) [14].

Coating the implant surface with polysaccharides is one of several strategies to improve bioactivity and simultaneously reduce bacterial infections [72,73]. Polysaccharides are a class of natural polymers with a broad spectrum of biological activities, including antioxidant, immunomodulatory, anticoagulant, anticancer, anti-inflammatory, antibacterial, and antiviral activity [74,75,76,77].

**Figure 9:**
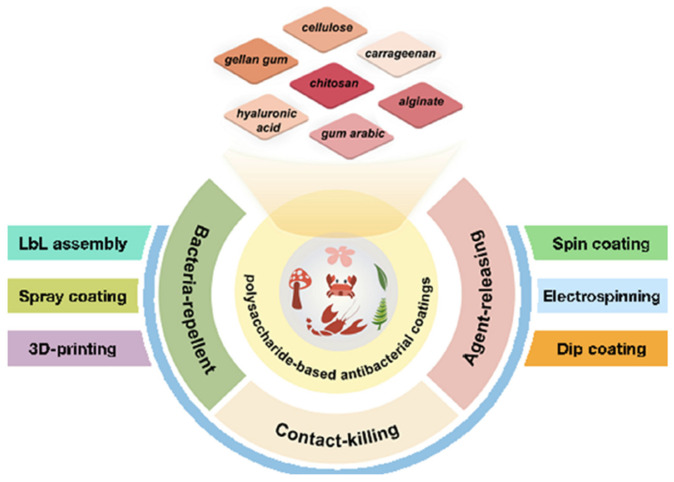
Illustration of polysaccharide-based antibacterial coating technologies: main types of polysaccharides, strategies for killing bacteria, and typical fabrication methods [74].

Therefore, polysaccharides are considered a good choice for the investigation of antibacterial materials (Figure 9) [74]. The antibacterial activity of polysaccharides is based on increasing the cell wall and cell membrane permeability and blocking DNA transcription and mycoprotein expression. Furthermore, they inhibit pathogen attachment to host cells and block nutrient or energy transport [78,79,80,81,82,83,84].

The aim of this article is to review the current status of the identification, functionalization, characterization, and application of bioactive polysaccharides derived from natural sources for dental implant applications. Research on biomedical applications, such as tissue engineering and the antibacterial activity of polysaccharides, is highlighted. The aim is to pave the way for the development of new strategies for tailored functional materials with potential application of polysaccharides from natural sources in implantology.

## 2. Methods

This review aims to provide an overview of relevant polysaccharides with antibacterial properties for application in dental implantology. The authors discussed the topics that can be included in this review. The group then decided to select the relevant literature to answer the following questions: Can polysaccharides combat the pathogens in implantology without triggering immune reactions and/or inflammation or altering the properties of human cells? Can understanding the antibacterial mechanism of polysaccharides support research in developing new strategies to discover new polysaccharides or improve their properties for applications in dental implantology? Is it possible to functionalize polysaccharides to possess antibacterial and osteogenic properties, two factors that are crucial for the success of implants in clinical practice?

**Figure 10:**
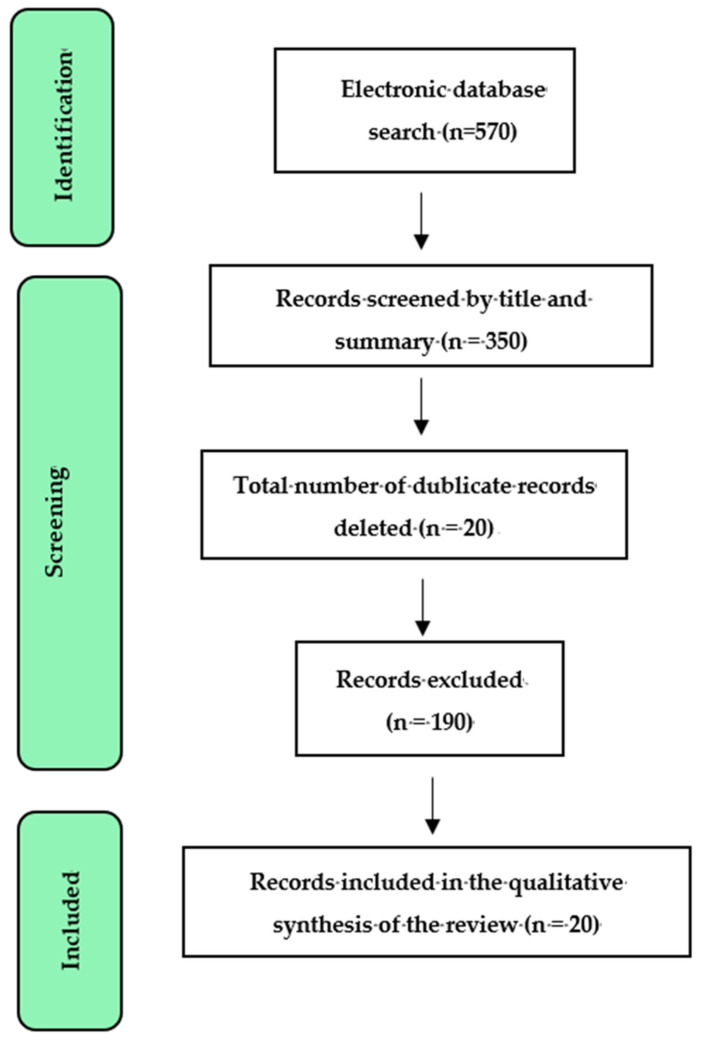
Study selection process of the literature search according to the PRISMA flow diagram.

The electronic databases PubMed, Medline, ProQuest, and Google Scholar were used to search for peer-reviewed scientific publications published between 2018 and 2025 using terms such as polysaccharides, biocompatible, antibacterial, osseointegration, implant, and bioactive materials. The selection of articles was performed according to the PRISMA flow diagram (Figure 10). Regarding the type of research, the studies had to be experimental in vitro. Animal and human studies were excluded.

## 3. Polysaccharides

**Figure 11:**
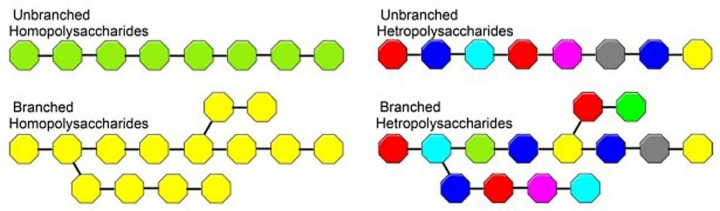
Branched and unbranched homopolysaccharides and heteropolysaccharides. Different monosaccharides are represented by different colors [86]. Reprinted with the permission of Springer Nature.

Natural polymers are often preferred to synthetic polymers because they are abundant in nature, easily accessible, and chemically modifiable. Polysaccharides are polymeric carbohydrates composed of more than ten glycosidic linked monomers. They are divided into two classes: homopolysaccharides (containing only one type of monomer) and heteropolysaccharides (containing more than one type of monomer). This is illustrated in Figure 11. Each polysaccharide source exhibits different branching chains, monosaccharide contents, molecular weights, and structural conformations. Depending on the type of linkage, polysaccharides can be classified into proteoglycans, glycoproteins, glycolipids, and glycoconjugates [85,86]. These are sugar residues that are linked glycosidically to each other or covalently to other structures, such as peptides, amino acids, or lipids [87,88]. Starch, glycogen, and cellulose are typical examples of homopolysaccharides. Arabinoxylans, glucomannans, and hyaluronic acid are examples of heteropolysaccharides with two or more different types of sugar residues in their polymer structures [85].

**Figure 12:**
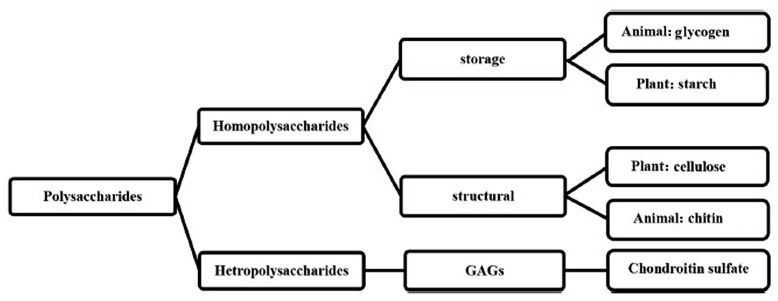
Classification of polysaccharides according to the type of monosaccharide building block and their physiological properties [86]. Reprinted with the permission of Springer Nature.

Polysaccharides have storage properties like starch or structural properties, e.g., cellulose, which provides physical structure and stability (Figure 12).

They can also be classified based on polyelectrolyte into positively charged polysaccharides (chitin, chitosan) and negatively charged polysaccharides (alginate, heparin, hyaluronic acid, pectin, chondroitin sulfate). Heparin, heparan sulfate, hyaluronan, chondroitin sulfate, dermatan sulfate, and keratan sulfate are the most important GAG polysaccharides in mammalian tissues. Glycosaminoglycans (GAGs) are the primary components of the cell surface and the extracellular matrix (ECM) [74,84,86].

Polysaccharides are the most abundant biological macromolecules in nature and are produced by animals, plants, and microorganisms. Marine polysaccharides have attracted the attention of researchers because of their abundance, bioactivity, structural diversity, low toxicity, and few side effects. They originate from diverse marine environments, including the Arctic Ocean (by microorganisms under extreme conditions), the Atlantic Ocean (sargassum, red algae, and marine actinomycetes), the Indian Ocean (coral reef ecosystems and sulfated seaweed polysaccharides), and the Pacific Ocean (kelp, wakame, shrimp, and crabs, which produce compounds such as fucoidan and chitosan). Unlike terrestrial animal and plant polysaccharides, marine polysaccharides exhibit distinctive structural features. Due to their extreme conditions, such as high pressure, high salinity, low temperatures, and limited light, marine polysaccharides are more highly sulfated than their terrestrial counterparts, resulting in higher charge density. They exhibit higher molecular masses and clearly defined, predictable structural motifs than terrestrial polysaccharides [88,89].

Polysaccharides are biopolymers that exhibit high biocompatibility due to their similarity to the human extracellular matrix and are therefore widely used for pharmaceutical purposes, encapsulation, gene therapy, wound healing, tissue engineering, and anti-inflammatory applications [89,90,91,92]. Polysaccharides can interact with and influence the immune response. Therefore, they play an important role in the treatment of many human diseases [93]. They exhibit specific antibacterial activity against both Gram-negative and Gram-positive bacteria. They are nontoxic and offer great potential for use as novel antibacterial agents in medicine and the food industry [94,95,96,97,98,99,100,101].

The bioactivity of polysaccharides and their physicochemical properties (solubility, fluid capacity) are influenced by numerous factors, such as structural conformation (type of linkage and degree of branching), molecular weight and its distribution, functional groups, monosaccharide compositions, monosaccharide linkages, and substituents [97,102,103]. Low-molecular-weight polysaccharides typically exhibit higher antibacterial activity because they penetrate bacteria more effectively, thus affecting their cellular proteins and energy metabolism. By reducing molecular weight, more reactive groups are exposed to the environment, which promotes diffusion and thus improves the bioactivity of the polysaccharides [104].

Sulfation, carboxymethylation, phosphorylation, acetylation, and other chemical modifications of polysaccharides can alter them and enhance their antibacterial activity. Sulfonation, in particular, is an effective means of enhancing the antibacterial activity of polysaccharides. Its mechanism of action may involve sulfate groups that enhance the disruption of bacterial cell walls and membranes, thus leading to bacterial death [92,101,105].

Cell membrane proteins, such as enzymes, carrier proteins, participants in the electron transport chain, and other proteins, play an important role in maintaining membrane permeability and integrity. Damage to membrane proteins can compromise the integrity of the enzyme system in the bacterial membrane and subsequently lead to bacterial death [78]. Fluidity is one of the fundamental properties of the cell membrane that is necessary for cell function and is primarily influenced by the phospholipid bilayer of the cell membrane. The antibacterial mechanism of polysaccharides is based on the suppression of fungal protein expression through the influence of fungal protease activity. This modulates enzyme distribution, conformation, and cofactors, increases bacterial cell permeability, and impedes nutrient transport [93]. The cell wall performs important functions, such as maintaining cell shape, participating in substance transport, and facilitating information transfer. Damage to the cell wall can lead to cell death [75,79].

**Figure 13:**
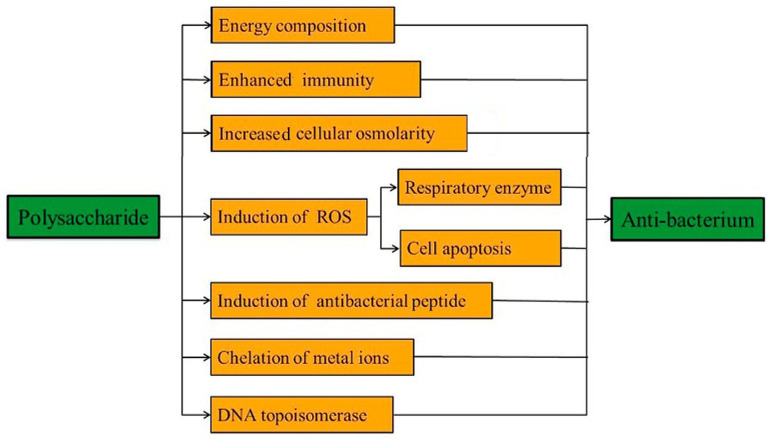
Antibacterial mechanisms of polysaccharides [78]. Reprinted with the permission of Elsevier.

Polysaccharides can bind to bacterial DNA targets and affect their replication, transcription, and translation. They thereby inhibit the synthesis of bacterial nucleic acids and proteins. Polysaccharides can also bind to plasmid DNA and cause its degradation into smaller fragments. This suggests that plasmid DNA could be a potential mechanism of action for polysaccharides with antibacterial effects (Figure 13) [77,78].

The current research focuses on the modification of native bioactive polysaccharides with anti-adhesive, bactericidal, and osteogenic functions to expand their applications in tissue engineering, bacterial and viral diseases, controlled drug delivery and release, wound healing, and wound care [98,99,100,101,102,103,104]. In the following, this review focuses exclusively on antibacterial bioactive polysaccharides and their modifications for use in dental implantology.

### 3.1. Antibacterial Polysaccharides

#### 3.1.1. Chitosan

Coating implant surfaces with materials that exhibit important properties, such as biocompatibility, biodegradability, osseoconductivity, accelerated wound healing, anti-inflammatory, and antibacterial properties, has a significant impact on the success of implant treatments. Among the materials that combine these essential properties, chitosan emerges as a promising candidate. Its unique combination of the above-mentioned properties makes chitosan a highly valuable material for improving the efficacy of implant treatments [103,106,107,108].

**Figure 14:**
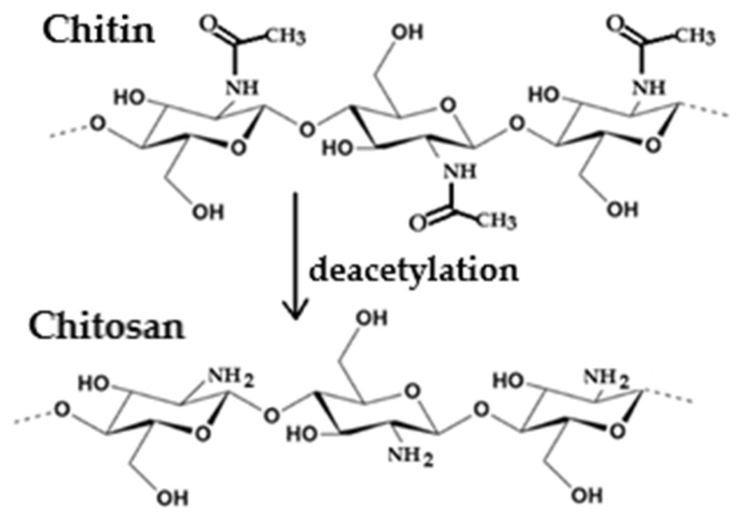
Schematic representation of the complete deacetylation of chitin to chitosan [104].

Chitosan, β-(1,4)-2-amino-2-deoxy-D-glucose, is a polysaccharide derived from chitin. It is a linear polymer found in the exoskeletons of crustaceans and insects and in the cell walls of fungi and plankton. Chitosan is produced from chitin by a partial or complete deacetylation process (Figure 14) [106,107]. The degree of deacetylation influences the antibacterial activity of chitosan, as the number of free amino groups increases with a decreasing number of acetyl groups.

**Figure 15:**
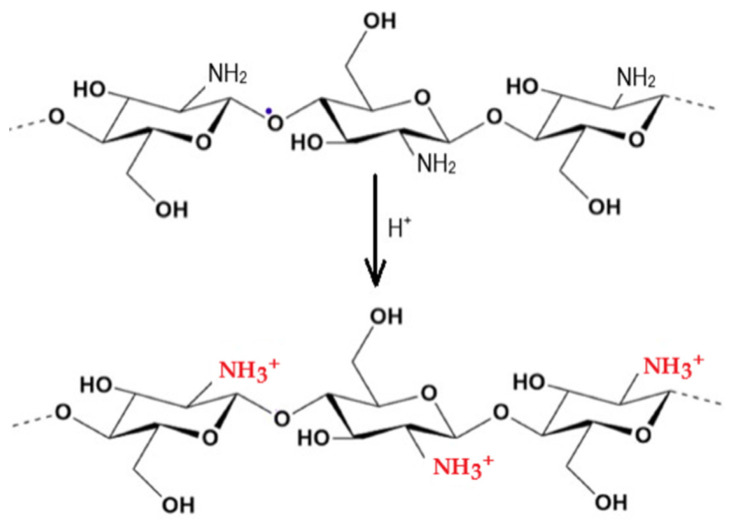
Schematic representation of the chitosan structure before and after acidification. NH_3_^+^ is the active functional group of chitosan [107].

The free amino group of chitosan is able to disrupt the growth of Gram-positive and Gram-negative bacteria. There are two suggested modes of action for the antibacterial activity of chitosan. The first one can be understood in terms of the positively charged amino group NH^3+^ (see Figure 15), which can bind to the negatively charged surface of bacteria under acidic conditions.

**Figure 16:**
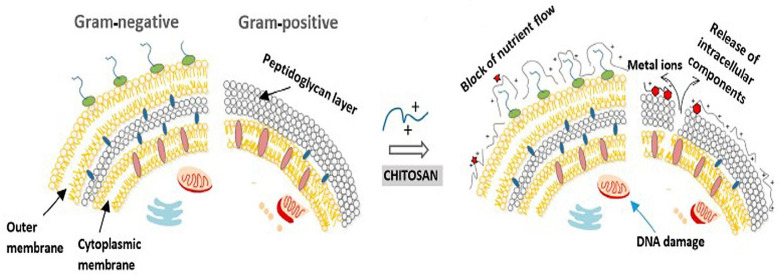
The antibacterial mechanism of chitosan on Gram-positive bacteria and Gram-negative bacteria [110].

The uniform charge distribution on the bacterial wall is disrupted, and the cell wall synthesis is impaired or even destroyed. This interaction leads to significant changes in the cell wall by altering membrane permeability, which, in turn, leads to an osmotic imbalance and release of intercellular components, triggering cell death. The depolarization of the cell membrane can induce Ca^2+^ uptake via the voltage-gated Ca^2+^ channel of the plasma membrane or other unknown transport channels, resulting in an increase in intracellular reactive oxygen species and cell apoptosis [78,102,104,106,107,108,109,110,111,112,113,114,115,116,117,118,119,120,121,122,123,124]. The second proposed mechanism of action for chitosan is that after being adsorbed by bacteria, it can penetrate porous cell walls and subsequently enter into bacteria. Chitosan can form a stable complex with DNA and disrupt DNA or RNA synthesis, thereby inhibiting bacterial proliferation (see Figure 16).

The antibacterial activity of chitosan depends on various factors. Intrinsic factors include its origin (crustaceans, insect shells, or fungi), concentration, molecular weight, and degree of polymerization. External factors affecting antibacterial activity include the pH of the environment and the type and sensitivity of the target microorganisms [106,109,110,111]. Chitosan exhibits its antibacterial activity only in acidic environments because it is poorly soluble in media with a high pH (pH ≥ 7). At a pH value below 6.5, chitosan molecules are protonated due to the high density of -NH^3+^ groups formed under such conditions (see Figure 15). This imparts a positive charge to the polymer and increases the intermolecular electric repulsion, resulting in a polycationic macromolecule. The decrease in pH is accompanied by the adsorption of chitosan to the bacterial surface [111].

The lower molecular weight, smaller size, and conformation of chitosan appear to be of fundamental importance, as the mobility is higher and small chains can be more easily attracted and interact ionically than larger ones. This promotes more effective binding of chitosan to the bacterial membrane surface [111].

**Figure 17:**
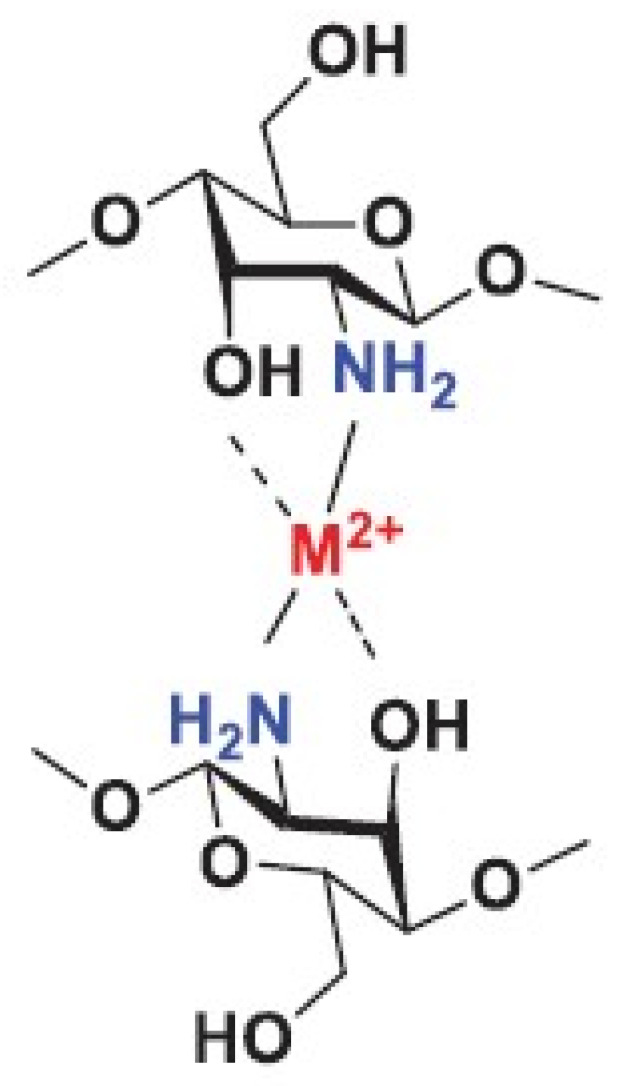
Schematic representation of the ability of chitosan to form chelates with metal ions [108].

Another antibacterial mechanism of chitosan is the chelation of metal ions, which inhibits bacterial growth (see Figure 17) [106,107,108]. Chitosan possesses excellent metal-binding properties due to its amino groups. Gram-positive and Gram-negative bacteria exhibit remarkable differences in their cell wall structure, where Gram-positive bacteria have thicker peptidoglycans while Gram-negative bacteria are enriched in lipopolysaccharide (LPS) [24,106,108]. The phosphate groups of teichoic acids in the peptidoglycan layer of Gram-positive bacteria particularly attract Mg^2+^ and Ca^2+^ cations, thus supporting the maintenance of enzymatic functions and membrane integrity. However, the elimination of the teichoic acid biosynthesis pathway in *S. aureus* leads to increased resistance to chitosan. This behavior indicates that the antibacterial activity of chitosan is more complex than simple electrostatic interactions [110]. Lipopolysaccharides, as components of the cell surface of Gram-negative bacteria, possess a negatively charged cell membrane and a strong affinity for divalent cations. Chelating agents can remove these cations, making the cell wall more permeable and less effective [109,110,111]. The interaction between the amino groups of chitosan and the divalent ions in the bacterial cell wall (such as Ca^2+^ and Mg^2+^) prevents the formation of toxins and inhibits bacterial growth. Chelation of vital metal ions thus deprives bacteria of nutrients and subsequently inhibits their growth [108,111].

In addition, chitosan can form a layer on the surface of bacteria, preventing other nutrients from entering the bacterial cells. Chitosan may be able to block oxygen transport and, consequently, inhibit the growth of aerobic bacteria [109,110,111,113,114,115].

#### 3.1.2. Pectin

**Figure 18:**
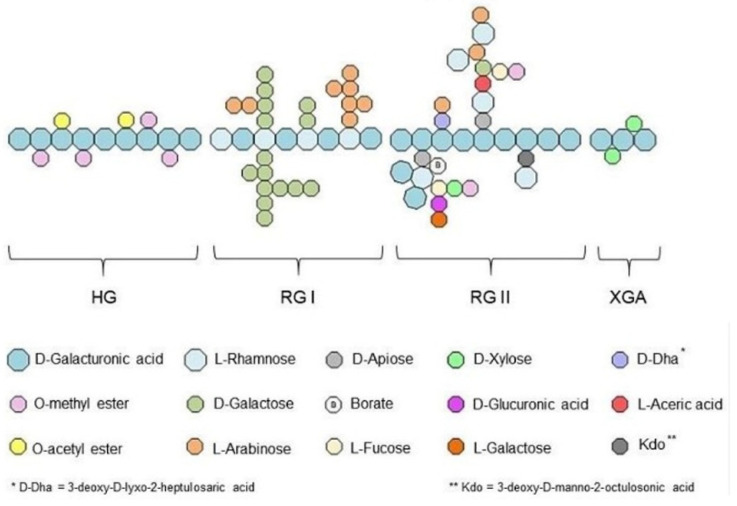
Chemical structure of the pectin molecule [125].

Besides chitosan, pectins are an interesting class of polysaccharides for dental applications. Pectins are heterogeneous polysaccharides with three main domains: an α-(1,4)-linked linear homo-galacturonic backbone (HG) alternating with two types of highly branched rhamnogalacturonans regions and the so-called RG-I and RG-II. RG-I is substituted by side chains of arabinose and galactose units. RG-II has a highly conserved structure and consists of an HG backbone branched with eleven different monosaccharides. This is shown schematically in more detail in Figure 18 [125].

Pectin, a plant polysaccharide, strengthens the cell walls of higher plants. Its composition is similar to the polysaccharides of the mammalian extracellular matrix, thus facilitating cell adhesion. Pectin is a negatively charged polysaccharide that reacts with the positive charge of chitosan through intramolecular electrostatic attraction, forming a complex polyelectrolyte structure. Conjugation of these biopolymers through a chemical reaction can enhance their antibacterial activity. Pectin itself exhibits antibacterial properties at a pH value lower than 5. The antibacterial activity of pectin depends on the amount of negatively charged carbonyl groups, which has a significant impact on bacterial viability due to changes in the zeta potential. This, in turn, leads to changes in the cell envelope composition and bacterial metabolism [125].

Pectin has a diverse molecular structure and is highly susceptible to physical, chemical, and enzymatic modifications. The various functional groups in the pectin structure can stimulate different functionalities. Certain modifications open up new applications for this polysaccharide by altering its physicochemical properties, such as formal charge, degree of esterification, and molecular weight. The class of modified pectins has a broader range of applications than native pectins, e.g., in cancer therapy and wound healing or as a bactericide [125,126,127,128].

**Figure 19:**
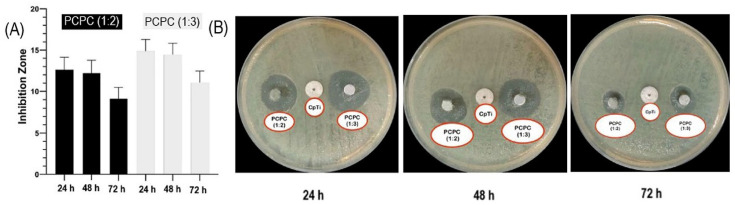
(**A**) Graphic illustration and (**B**) CpTi disc representation of the IZD (inhibition zone diameter in mm) of the antibacterial test of investigated groups over 24, 48, 72 h [129].

Alsharbaty et al. investigated the antibacterial properties of a mixture of chitosan (extracted from shrimp shells; ≥ 75% deacetylated; average M_w_ 190–310 kDa), pectin (extracted from citrus peel, galacturonic acid ≥ 74% dried basis, average M_w_ 485 kDa) in a ratio of 2:1 and (3:1) and PVA to form PCPC. PVA (polyvinyl alcohol) is a nontoxic, biocompatible, and biodegradable polymer [129], and PCPC is the chitosan–pectin polyelectrolyte polymer. The nanoparticle mixtures were divided into two groups: PCPC (1:2) and PCPC (1:3). Commercially available pure Ti (Cp Ti) discs were used as the substrate, which were coated with the aforementioned PCPC mixtures by the electrospinning/spraying method. As observed in the graph in Figure 19A and the images of the samples in Figure 19B, both PCPCs showed antibacterial activity against anaerobic bacteria, with PCPC (1:3) being superior to PCPC (1:2) (Figure 19).

As shown in Figure 19, the antibacterial activity of both groups decays with increasing exposure time. This can be explained by the reduction of positively charged groups in chitosan due to the reaction with the negatively charged groups of the bacteria derived from humans with peri-implantitis. To enhance the long-term antibacterial activity of the chitosan–pectin polyelectrolyte polymer, the combination of chitosan with other pectins, such as lemon IntegroPectin, is recommended (Table 1).

According to Alsharbaty et al., the PCPC (1:3) samples exhibited higher biocompatibility compared to the other groups, while the CpTi samples showed the lowest biocompatibility [129]. Leaching of metallic ions into the surrounding biological environment of cells can cause oxidative stress, inflammation, and cell mortality [129]. Therefore, it is important to coat the CpTi surface with a biocompatible material such as PCPC (1:3) polyelectrolyte polymer. The cellular toxicity response is influenced by various factors, such as the particle size, shape, surface charge, material concentration, composition, and geometry of nanoparticles [140,141,142,143,144,145].

For clinical applications, it is important to conduct experiments over a longer period of time. A three-day period is very short to evaluate this mixture for application in dental implantology. Furthermore, it would be very interesting to investigate the osseointegration properties of this mixture for clinical applications.

Coating titanium implants with polysaccharides such as chitosan offers the possibility of protecting the implant from a range of microorganisms, including anaerobic bacteria [146]. A coating with PCPC in a ratio of 1:3 was found to be more stable than a PCPC layer in a 1:2 ratio. This can be explained by the high concentration of chitosan [129]. The important factors for implant coating include uniformity, thickness, stability, biocompatibility, low cytotoxicity, and the potential for optimization of the mechanical properties to withstand physiological conditions.

From this study, it can be concluded that the mixture of chitosan, pectin, and PVA (PVA improves the spinnability process) represents a promising and feasible coating method for dental implants made of commercially pure titanium.

It is important to note that antibacterial efficacy depends not only on the chosen coating materials but also on the surface texture of the implant and the coating process itself. A textured surface and a suitable coating process can promote integration between bone and titanium implants, improving the contact at the direct interface. Nanostructured surfaces can facilitate bone cell attachment, proliferation, and differentiation, thus improving the antibacterial properties of implant surfaces [145,146,147,148].

#### 3.1.3. Alginate

**Figure 20:**
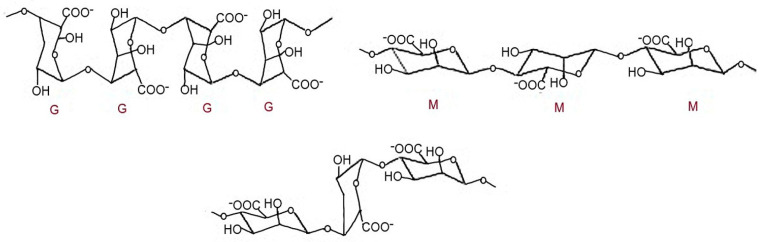
Schematic representation of the chemical structure of alginate with different distributions of M and G monomers. GGG (G-block), MMM (M-block), and MGM/GMG (alternating block) [149].

Alginates are anionic polysaccharides. Alginic acid is a component of brown algae and makes up about 40% of their dry mass. Alginate is a linear polymer composed of d-mannuronic acid blocks linked by β-1,4 (M) and l-guluronic acid blocks linked by α-1,4 (G), as schematically shown in Figure 20 [149].

Alginate is a natural polymer widely used in drug and protein delivery systems. Chemically, alginate consists of randomly copolymerized blocks of β-1,4-D-mannuronic acid (M) and α-1,4-l-guluronic acid (G), which are linked linearly via 1–4 glycosidic bonds. The polymer consists of consecutive G-blocks (G-G-G), consecutive M-blocks (M-M-M), and alternating M and G-blocks (M-G-M) [100,149,150].

The intracellular matrix contains alginate in the form of a gel containing ions such as Na^+^, Ca^2+^, Mg^2+^, Sr^2+^, and Ba^2+^. The ability of alginate to form a gel depends on its weight, molecular structure, and the concentration of the gelling agent. The physical and chemical properties of alginates are determined by the arrangement of individual monomers in the chain and their molecular weight. Alginate exhibits high absorbency and antibacterial properties and can accelerate wound healing. It exhibits excellent biocompatibility and nontoxicity. The use of alginate can modify the physicochemical behavior of drugs, thus improving their efficacy and safety in drug delivery systems. Due to their antioxidant properties, antibacterial activity, porosity and gelling ability, they are used in tissue engineering for tissue improvement [138,149,150,151,152,153,154].

Duan et al. investigated the properties of chitosan–sodium alginate to improve the corrosion resistance and antibacterial activity of titanium surfaces [152]. Implants and dental crowns are generally designed for long-term durability, but titanium and its oxide layer can be destroyed by saliva during prolonged use, thereby shortening the implant’s lifespan. To prevent implant corrosion, the bioactive coating of titanium has attracted considerable research interest as an effective surface modification [147].

Duan et al. used Ag-doped chitosan (CHI) and Na alginate (SA) polyelectrolytes to fabricate a self-reinforcing coating on a PDA (polydopamine)-coated titanium substrate in a layer-by-layer process. SA was extracted from seaweed because of its low cost, nontoxicity, chemical inertness, excellent biocompatibility, good film-forming, and anti-corrosive properties. The PDA-coated titanium surface was used to prepare AgNPs (silver nanoparticles) from a AgNO_3_ solution [152].

**Figure 21:**
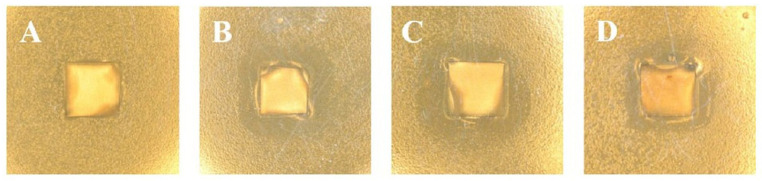
Inhibition zones of bare Ti (**A**), AGNPs/PDA/Ti (**B**), CHI/AGNPs/PDA/Ti (**C**), and SA/CHI/AGNPS/PDA/Ti (**D**) [152]. Reprinted with the permission of Elsevier.

**Figure 22:**
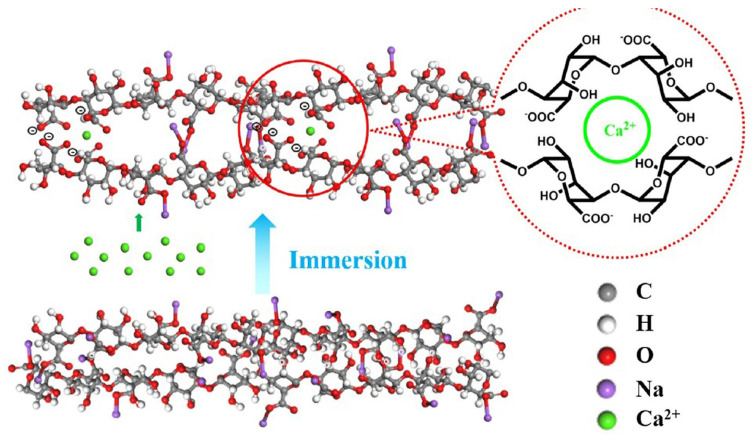
Schematic illustration of the possible self-strengthening mechanism of the SA/CHI/AGNPS/PDA/Ti coating in fluorine-containing artificial saliva [152]. Reprinted with the permission of Elsevier.

Figure 21 shows the antibacterial activity of the coated substrates (PDA/Ti, AgNPs/PDA/Ti, CHI/AgNPs/PDA/Ti, and SA/CHI/AgNPs/PDA/Ti), with untreated Ti as a reference against *S. mutans bacteria.* Compared to untreated Ti, all the other samples showed significant inhibition zones against *S. mutans*. The diameter of the inhibition zone for AgNPs/PDA/Ti was the largest, while the diameter of the inhibition zone for spinning chitosan and adsorbed sodium alginate gradually decreased. The authors also investigated the cell proliferation in untreated Ti and SA/CHI/AgNPs/PDA/Ti samples. A significant increase in the cell counts was observed for the SA/CHI/AGgPs/PDA/Ti samples. The coating showed no negative impact on cell proliferation because the release of Ag ions was controlled by CHI and SA. The authors attributed the antibacterial activity of all the samples to the presence of Ag^+^ ions, which bind to the thiol groups of bacterial proteins and can interfere with DNA replication. However, they could not explain the role of chitosan and Na alginate in the antibacterial activity of the samples. The best results in the corrosion inhibition tests were shown for the CHI/AgNPs/PDA/Ti and SA/CHI/AgNPs/PDA/Ti samples [152]. The SA/CHI/AgNPs/PDA/Ti samples showed excellent self-reinforcing activity in fluorine-containing artificial saliva. The surface of the SA/CHI/AgNPs/PDA/Ti sample appeared gelatinous after different immersion times due to the reaction between SA molecules and Ca^2+^ cations from corrosive artificial saliva. The possible self-reinforcing mechanism is schematically shown in Figure 22.

During immersion, Ca alginate gels (CA) were formed because of the strength and specific interactions between the G units of the SA and the Ca^2+^ cations in the saliva solution [152].

Ca^2+^ ions can act as cross-linkers in corrosive media to coordinate two successive G units of two pairs of different chains, thus continuously improving the corrosion protection performance of the coating. The formation of CA builds a stable barrier layer, thus ensuring a long-term self-reinforcing effect in fluorine-containing artificial saliva [152]. The development of a self-reinforcing coating with antibacterial and osseointegrative properties is currently the focus of research.

**Figure 23:**
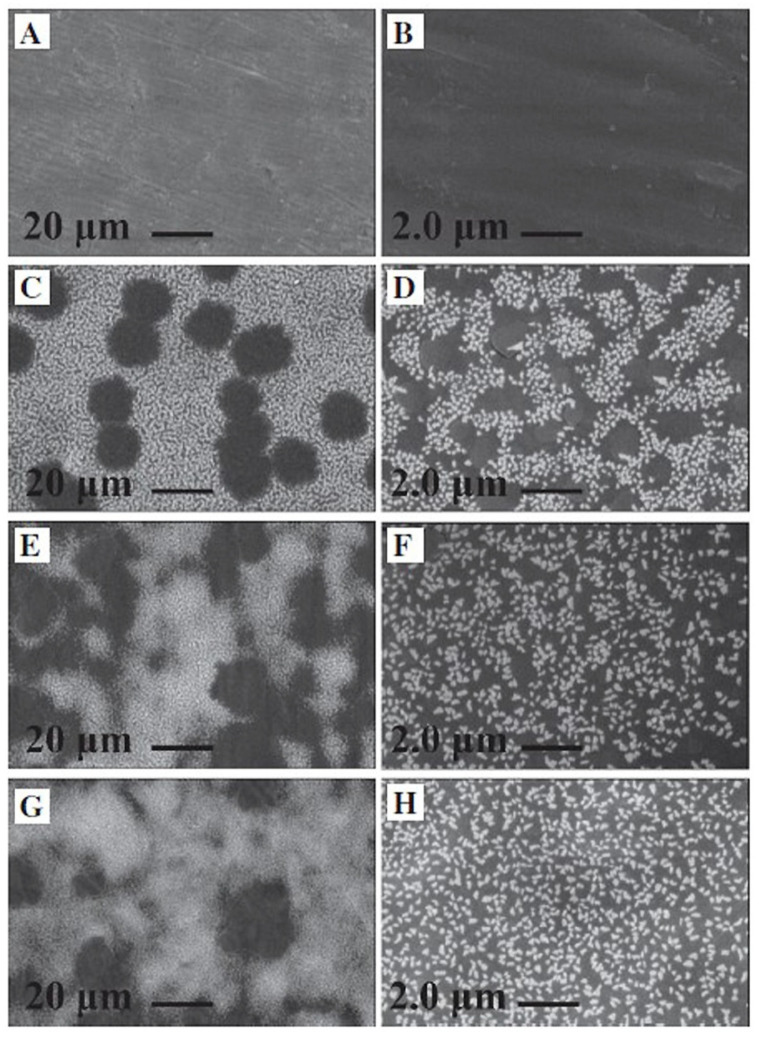
SEM images of the untreated Ti surface (**A**,**B**), coat 1 (**C**,**D**), coat 4 (**E**,**F**), and coat 8 (**G**,**H**) [154]. Reprinted with the permission of de Gruyter.

Vakili et al. [154] investigated the antibacterial activity of a mixture of chitosan (0.5%) and alginate (0.5%) on titanium plates (0.1 × 10 × 10 mm). The titanium plates were coated with the polymers by spin coating at speeds of 1000 rpm (coat 1), 4000 rpm (coat 4), and 8000 rpm (coat 8). The resulting surface structures are shown in Figure 23.

The surface structures shown in Figure 23 illustrate that the homogeneity in the deposited coating strongly depends on the spinning process conditions. The best coating was achieved at a spinning speed of 8000 rpm. The interpenetrating polymer network of chitosan and alginate, which forms during the reaction of positively charged chitosan and negatively charged alginate, reduces the degradation rate of alginate and increases its stability [154].

During storage of the coat 8 samples for 7 and 14 days in an SBF (simulated body fluid) solution, hydroxyapatite formed on the sample surface. This can be seen in the higher magnification inset in Figure 23D. The chitosan–alginate mixture possesses bioactive properties that are of great importance for the bonding of the implant to bone and the stability of implant integration. The antibacterial activity of the coat 8 samples increased from 4.1% for untreated titanium to 36.31%, associated with a reduction in CFU from 2.18 × 10^6^ to 1.62 × 10^6^. Vakili et al. attributed this strong rise in the antibacterial activity of the coat 8 samples to the presence of chitosan in the mixture [154]. No cytotoxicity was observed on the titanium coated with the chitosan–alginate polymer, indicating the biocompatibility of this polymer mixture.

**Figure 24:**
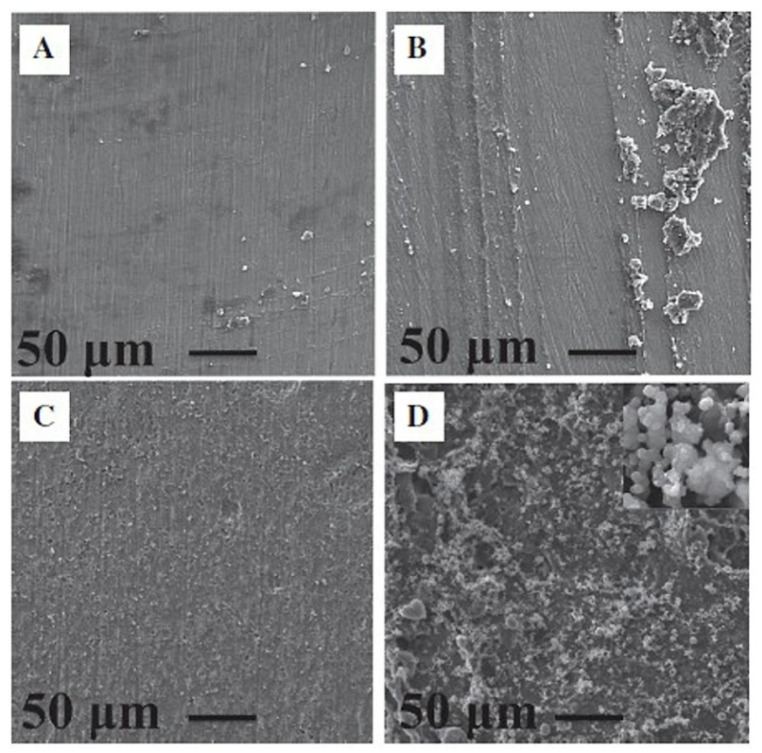
SEM images of untreated Ti after (**A**) 7 days and (**B**) 14 days and of coat 8 after (**C**) 7 days and (**D**) 14 days of immersion in an SBF solution [154]. Reprinted with the permission of de Gruyter.

The adhesion and growth of L929 cells on the coated titanium surface confirmed the bioactivity of this polymer blend (Figure 24) [154]. The bioactivity of the chitosan–alginate polymer can be explained by the intrinsic properties of chitosan. Chitosan and fibroblast cells have a positive and negative charge, respectively, which promotes cell adhesion to the chitosan surface. The high permeability of oxygen inside alginate makes the alginate structure a highly suitable substrate for cell adhesion and subsequent growth [138,154]. In summary, the chitosan–alginate blend possesses two very important properties for implantology: it is bioactive and simultaneously antibacterial. This represents the future of material design in dentistry.

#### 3.1.4. Chitosan Nanoparticles

Chitosan nanoparticles have a wide range of applications in medicine and pharmaceutics. Nanostructured chitosan particles are smaller than 100 nm in at least one of their dimensions. They enable enhanced interactions between components, making them more suitable for the restoration and repair of human tissue, and as carriers in drug and gene delivery systems [80,118,141,143]. In their crystalline forms, the surface of chitosan and nanochitosan can be modified with various ligands. This includes inorganic ions and hydrophobic or hydrophilic compounds. They can also be functionalized with advanced molecules, such as antibodies, proteins, peptides, polysaccharides, and nucleic acids, which are covalently bound to the primary molecule or serve as carriers for these molecules through nanoencapsulation. Nanochitosan displays high surface-to-volume ratios. The properties of chitosan nanoparticles are rather broad. They are biocompatible and nontoxic and can be used for encapsulation and chain refinement of drugs and active ingredients. They prevent the enzymatic degradation of drugs and reduce damage to non-targeted tissues or cells. This makes them of great use in drug delivery, cancer treatment, and biological imaging and diagnosis [119,120]. CSNPs have antibacterial activity against *E. faecalis, S. mutans, A. actinomycetemcomitans*, *P. gingivalis*, and *C. albicans* [141,142,143].

According to Alhomrany et al., CSNPs exhibit cytotoxicity depending on the particle size and concentration. The electrostatic interaction between the positive charges of the amino group of CSNPs and the negatively charged cell membrane is responsible for this cytotoxicity effect. The small size of these nanoparticles probably allows them to penetrate the cell membrane, subsequently leading to cell death [142]. Other authors, such as Ibrahim et al., observed no cytotoxicity in their experiments and evaluated CSNPs as a biocompatible material. This discrepancy may be explained by the charge density of CSNP films prepared by electrospraying [141].

One problem with CSNPs is their stability [142]. If the van der Waals forces exceed the repulsive electrostatic force, the nanoparticles tend to agglomerate. The surface charge, concentration, size, structure, chemical composition, and organic components of the culture medium appear to be factors that contribute to the formation of large agglomerates [142].

Nanotoxicity and the potential human health risks of nanoparticles should be carefully evaluated [144,145]. Therefore, further studies are needed to assess the cytotoxicity of CSNPs.

Ju et al. investigated the possibility of increasing the antimicrobial activity of chitosan without compromising its biocompatibility [155]. In their study, they used polyvinyl alcohol (PVA) and bacterial cellulose (BC) to improve the mechanical properties and appearance of chitosan (CS) and chitosan nanoparticle (CSNP) films. The concentration of CS and CSNPs in the films was 0.5%, 1.0%, and 2.5% w/v. Antibacterial activity was tested using the agar plate method against *E. coli* and *S. aureus* bacteria.

**Figure 25:**
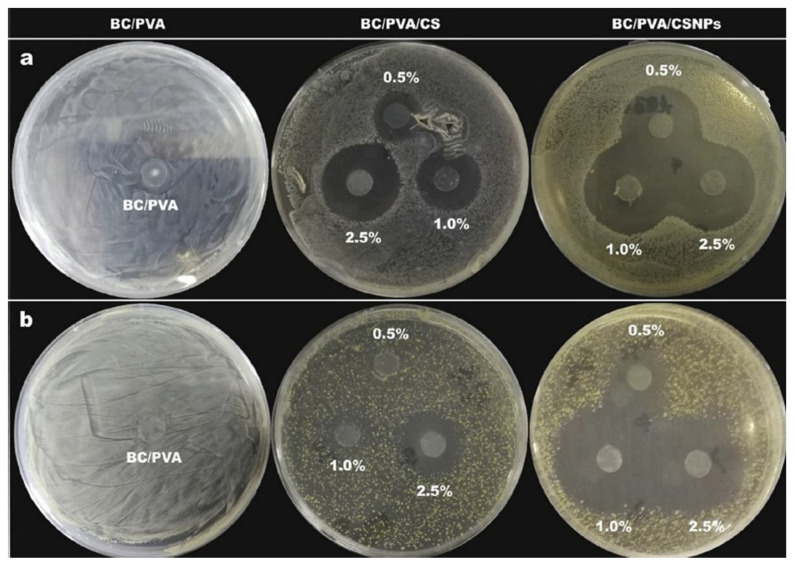
Antibacterial activity of CS and CSNPs at different concentrations (*w*/*v*) against (**a**) *E. coli* and (**b**) *S. aureus* [155]. Reprinted with the permission of Elsevier.

As shown in Figure 25, the BC/PVA films exposed to bacteria showed no antibacterial activity. The BC/PVA/CS films with 0.5% and 1.0% *w*/*v* CS showed low antibacterial activity against *S. aureus*, while they showed maximum antibacterial activity at a concentration of 2.5% *w*/*v* CS. While the antibacterial activity of CS-doped films strongly depends on the CS concentration, the BC/PVA/CSNPs films showed excellent antibacterial activity *against E. coli* and *S. aureus* regardless of the CSNPs concentration [155]. The higher antibacterial activity of CSNPs compared to regular CS can be attributed to their larger specific surface area, which leads to a higher positive charge density and provides more sites (referring to the amino groups; see Figure 15) for interaction with negatively charged bacteria [111,112]. Increasing the CSNP concentration did not lead to an increase in their antibacterial activity, as was the case with conventional CS. This may be due to the fact that the charge effect of CSNPs reaches a steady state in antibacterial activity even at a lower concentration. This is a well-known effect for nanoparticles, as their surface-to-volume ratio increases drastically compared to bulk materials.

Ju et al. did not investigate the cytotoxicity of their samples, even though it is an important factor for dental applications. The bioactivity of CSNPs compared to CS is also an interesting and relevant topic for implantology. However, the antibacterial activity of CSNPs against Gram-positive and Gram-negative bacteria opens up new possibilities for infection control in implantology.

#### 3.1.5. Carboxymethyl Chitosan

**Figure 26:**
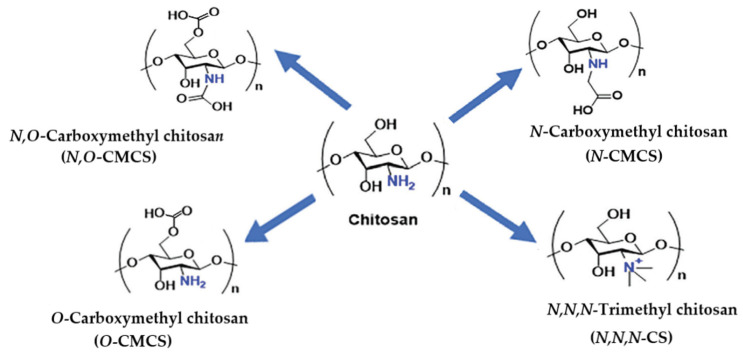
Chitosan and some of its derivatives [108].

Chitosan (CS) is a naturally derived cationic polysaccharide with physicochemical and biological properties suitable for tissue engineering and other biomedical applications. Its main disadvantage is its limited solubility in aqueous media at a neutral pH due to its rigid crystalline structure. This limits its effective use in various applications. The hydroxyl and amino groups of chitosan (see Figure 14) allow for the modification of its physical solubility and electrical charge. Carboxymethylation is a hydrophilic modification of chitosan. It is used to produce carboxymethyl chitosan (CMCS). CMCS has numerous biomedical applications, such as wound healing, bioimaging, tissue engineering, drug/gene delivery, and as a biosensor. Furthermore, it exhibits bactericidal, antifungal, antioxidant, antitumor, and anti-inflammatory properties [132,133]. CMCS is non-cytotoxic to fibroblasts, which is an important requirement for most materials in biomedical applications. CMCS exists in three different derivatives: *O*-carboxymethyl chitosan (*O*-CMCS), *N,O*-carboxymethyl chitosan (*N,O*-CMCS), and *N*-carboxymethyl chitosan (*N*-CMCS). These are schematically shown in Figure 26.

In contrast to *N*-CMCS and *N,O*-CMCS, *O*-CMCS possesses both -COOH and -NH_2_ groups, allowing for a wide range of modifications [108,133,135]. Of the three carboxymethyl chitosan derivatives mentioned above, *O*-CMCS exhibits superior antimicrobial activity due to the large amount of available -NH_2_ groups present. The presence of COO^-^ groups on the *O*-CMCS molecule and the formation of hydrogen bonds between *O*-CMCS chains and water are responsible for its improved solubility in aqueous solutions [131,133,134,135,136]. The incorporation of -CH_2_COOH groups into the polymer structure typically increases the viscosity and hydrodynamic volume, reduces toxicity, and improves the biocompatibility of the polymers. The amphoteric properties of CMCS polymers enable a direct response to pH changes through amine and carboxylic groups [131].

The antibacterial activity of chitosan and its derivatives against *E. coli* increases in the order N,O-CMCS < chitosan < N-CMCS < O-CMCS, which is due to the increase in the number of NH^3+^ groups starting from N,O-CMCS. A replacement in O-CMCS occurs only at OH groups. Thus, the number of amino groups remains constant, leading to the highest antibacterial activity for O-CMCS [108,133]. The antibacterial activity of CMCS can be explained by the positively charged amino groups, which enable interaction with negatively charged bacterial cell membranes, causing their disruption and subsequent cell death. In addition, CMCS macromolecules can penetrate bacterial cells and disrupt various processes, such as inhibiting enzyme activity or DNA synthesis. The antibacterial activity of chitosan, N,O-CMCS, and O-CMCS nanoparticles has been tested against *S. aureus.* It was found that the antibacterial activity of chitosan was lower than that of O-CMCS and N,O-CMCS nanoparticles [108,133].

By incorporating quaternary ammonium groups into chitosan, the polymer is permanently positively charged, making the CMCS derivative water-soluble regardless of the pH of the aqueous medium and simultaneously increasing its antibacterial activity [112,137,156].

Implant-related infections often occur within the first four weeks after surgery and peak at approximately four weeks. During this time, appropriate antibacterial materials are required to prevent and control infections. Towards the end of this period, osseointegration and secondary stability of the implants begin. They are crucial for improving the overall implant stability and thus for the ultimate success of the implant. The overall implant stability reaches a stable state approximately eight weeks after implantation and full stability within 3–6 months after implantation [157,158]. Maintaining an acceptable level of antibacterial activity to prevent biofilm formation is necessary. Therefore, effectively promoting osseointegration and antibacterial activity are extremely important [157,158,159]. The immobilization of enzymes, cytokines, or other components of the extracellular matrix (ECM) on biomaterial surfaces plays a crucial role in triggering specific cellular responses to strengthen the tissue–implant surface [157,158,159].

To meet the requirements for a stable implant, Lin et al. used a modified layer-by-layer (LBL) coating method to develop a multifilm structure with cross-linking via amido bonds [159]. The resulting structure was stable in tris-buffer and slowly degraded under the action of a collagenase solution. Lin et al. confirmed that their method can be used to develop antibacterial coatings with long-lasting, release-killing, and contact-killing properties. They used quaternary ammonium carboxymethyl chitosan (QCMC) as an antibacterial material. Due to its large number of carbonyl and amino groups, it is an ideal water-soluble antibacterial agent and is capable of forming a stable covalent multifilm [159]. The authors used collagen (COL) and hydroxyapatite (HAP) to mimic the extracellular matrix (ECM) of natural bone and induce osseointegration [159]. As a carrier material for the multifilm coating, Lin et al. used discs made of pure titanium, which is inert and does not have sufficient bioactivity.

**Figure 27:**
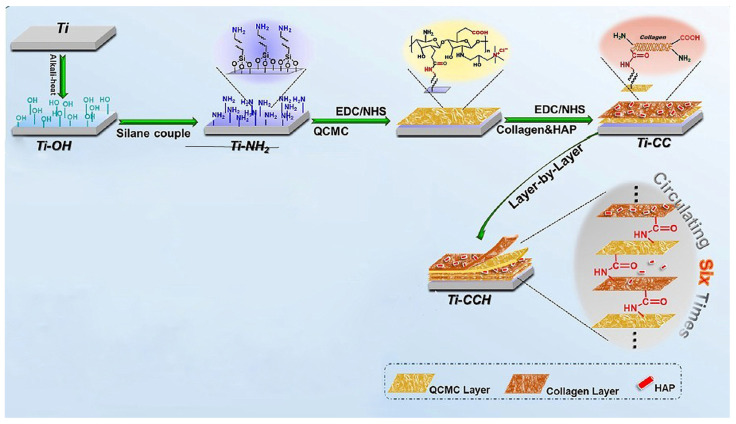
Schematic illustration of the processing sequence of Ti-CCH samples [159]. Reprinted with the permission of Elsevier.

Therefore, the authors treated the Ti surface with (a) hot alkali etching solution (Ti-OH), (b) silane + coupling agent (Ti-NH_2_), (c) reaction of Ti-NH_2_ with QCMC (Ti-CC), and (d) preparation of a multilayer film using LBL techniques to form stable bonds with QCMC, COL, and HAP (Figure 27) [159].

**Figure 28:**
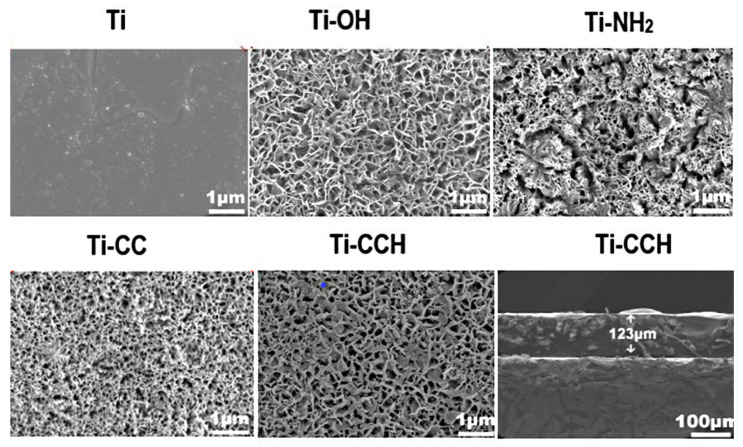
SEM images showing the structural morphology of Ti, Ti-OH, Ti-NH_2_, Ti-CC, and Ti-CCM and the thickness of the multifilm coating on Ti-CCH [159]. Reprinted with the permission of Elsevier.

As shown in Figure 28, all the samples exhibit different morphological microstructures. The Ti samples possess a smooth surface, which transformed into a palisade microstructure (Ti-OH) or a disordered 200–400 nm wide nano-grid microstructure (Ti-NH_2_) after treatment. After coating with macromolecules, a nano-grid microstructure with a thicker pore wall of 50–200 nm pore size and a layer thickness of 123 µm (Ti-CCH) was observed. The nano-grid structure created a large surface area and provided additional conjugation sites for biomolecules and cells, which positively influenced osteoblast adhesion and proliferation [159]. The deposition of HAP with a pore diameter of 200 nm and its uniform distribution over the substrate surface of Ti-CCH could mimic the natural bone microenvironment and induce osteoblast adhesion and proliferation [159].

Lin et al. used *S. epidermidis*, *E. faecalis,* and *S. aureus* to evaluate the antibacterial activity of the samples. Antibacterial contact and release activity were calculated using the plate spreading method.

**Figure 29:**
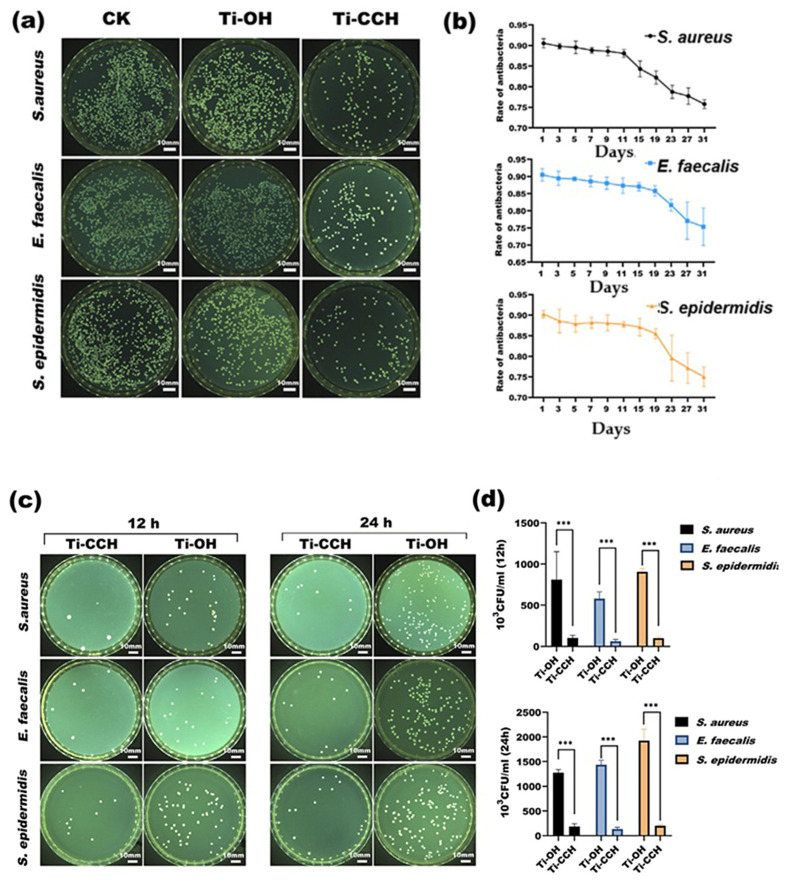
Antibacterial effect of Ti-CCH and Ti-OH in vitro. (**a**) Release-killing effect evaluated by the spread plate method. (**b**) Quantitative analysis of release-killing ratios against three bacteria. (**c**) Contact-killing effect evaluated by the spread plate method. (**d**) The number of viable bacteria adhering to the surface of Ti-OH and Ti-CCH after 12 h and 24 h co-cocultivation. *** *p* < 0.001 [159]. Reprinted with the permission of Elsevier.

As shown in Figure 29a,b, the rates of *anti-S. aureus*, anti-*S. epidermidis,* and anti-*E. faecalis* in the medium varied from an initial 90.5% to 75% in the first month [159]. This indicates that the QCMC in Ti-CCH can be sustainably released into the surrounding environment and eradicate most bacteria associated with a high risk of peri-implantitis. Even though some bacteria penetrated the antibacterial barrier and adhered to the implant surface, 85.2% to 89.4% of *S. aureus* and *S. epidermis*, as well as 90.6% of *E. faecalis*, were killed by the remaining QCMC on the bottom layers of the multifilm structures after 24 h, as shown in Figure 29c,d [159]. The antibacterial performance of Ti-OH was drastically inferior compared to the results observed for the Ti-CCH samples.

The antibacterial activity of QCMC can be explained by the presence of positively charged quaternary ammonium groups, which can bind to anionic bacterial membranes through electrostatic interaction, ultimately leading to membrane disruption and cytoplasmic leakage [22]. The binding mechanism itself is nonspecific, and most bacteria could be killed, thus preventing drug-resistant infection [159].

Lin et al. also investigated the osteogenic activity of Ti-CCH and Ti-OH and found that Ti-CCH exhibited a higher osteogenic activity than Ti-OH. Furthermore, Ti-CCH demonstrated excellent biocompatibility and did not affect the behavior of osteoblastic cells.

The long-lasting multi-antibacterial activity of the QCMC/COL/HAP coating, as well as its ability to promote bone formation and osteogenesis, are important findings that pave the way for further optimization in the development of scaffold materials and implant treatments.

#### 3.1.6. Xanthan Oligosaccharide

**Figure 30:**
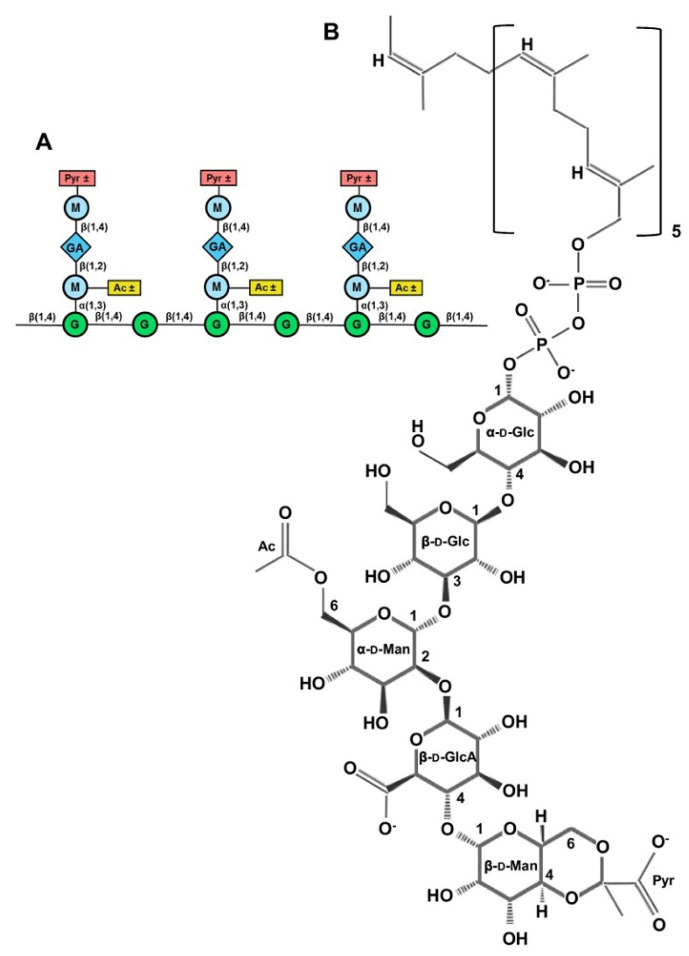
(**A**) Structure of a xanthan fragment. G, glucose; GA, glucuronic acid; M, mannose; Pyr, pyruvoylation; Ac, acetylation; ±, variable. (**B**) Acetylated and pyruvoylated xanthan monomer 4,6-CH3(COO-)C-d-Man-β-(1,4)-d-GlcA-β-(1,2)-6-O-acetyl-d-Man-α-(1,3)-d-Glc-β-(1,4)-d-Glc-α-1-diphospho-ditrans, octacis-undecaprenol [161].

Xanthan gum is a naturally occurring polysaccharide obtained from *Xanthomonas campestris*. The xanthan gum backbone consists of β-D-glucose linked to cellulose. Figure 30 shows the schematic structural configuration. The trisaccharide β-D-mannose (1-4)-α-D-glucuronic acid-(1-2)-α-D-mannose is linked to the O (3) position of every other glucose residue. Ketal bonds link pyruvic acid residues to approximately half of the terminal mannose residues. The terminal mannose residues also carry acetate groups [160,161,162].

Xanthan gum possesses many functional groups, such as carboxyl and hydroxyl groups. These groups can be modified or functionalized to expand the application of xanthan gum. The chemical structure of xanthan gum depends on the production parameters [161]. The properties of a xanthan gum solution depend on its chemical structure, the type of salt present in the medium, pH, and temperature. However, xanthan gum can be dissolved in both cold and hot water [161].

Xanthan gum is used as a thickening, stabilizing, or suspending agent in various applications, including the food, pharmaceutical, and cosmetic industries. Xanthan is chemically stable over a wide range of temperatures, pH values, and salt concentrations. Xanthan gum is not a popular polymer in bioremediation processes. It was used only as an additive to improve the properties of carriers or immobilized cells. The performance of xanthan gum relies on its macromolecular conformation and association in solution and at the interface. In water, xanthan gum can change from its helix arrangement into a random coil shape in response to stimuli caused by changes in pH, ionic strength, temperature, and shear force [162,163]. Ongoing research focuses mainly on the development of novel xanthan-based products and functional materials for biomedical and engineering applications [139,164]. Xanthan gum can be utilized in combination with other biopolymers to develop edible films and coatings that offer advantages over synthetic plastics in terms of biodegradability, safety, and production from renewable resources [163]. Composite films of xanthan gum and chitosan mineralized with hydroxyapatite are suitable for filling and repairing bone defects. Films impregnated with a solution of calcium hydrogen phosphate showed good results in in vitro cell adhesion tests [163]. Grafted-poly(N-vinyl imidazole) based on xanthan gum showed remarkable antibacterial properties against *E. coli* and *S. aureus* [161]. Oligosaccharides obtained from xanthan gum by partial hydrolysis can be used as antibiofilm agents [164].

Wang et al. investigated the antibacterial activity of a low-molecular-weight xanthan gum (LW-XG), produced by the endophytic fungus *Chaetomium globosum* CGMCC 6882 through the biodegradation of commercial xanthan, on *S. aureus* [139,164]. The monosaccharide composition of LW-XG was glucose, mannose, and glucuronic acid in a molar ratio of 1.63:1.5:1.0. The molecular weight of LW-XG was 4.07 × 10^4^ Da, which was significantly lower than that of commercial xanthan (2.95 × 10^6^ Da) [164]. The effect of LW-XG against the biofilm formation of *S. aureus* was evaluated by the crystal violet staining method.

**Figure 31:**
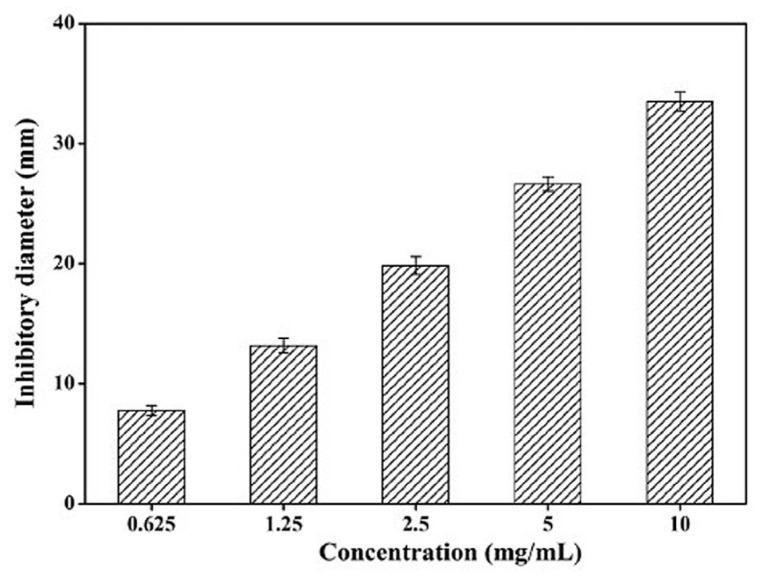
Inhibitory zone tests for different concentrations of LW-XG against *S. Aureus* [139]. Reprinted with the permission of Elsevier.

As shown in Figure 31, the antibacterial activity against *S. aureus* depended on the concentration of LW-XG. The highest inhibition zone of 33.51 ± 0.81 mm was observed at a concentration of 10.0 mg/mL, and the lowest inhibition zone of 7.75 ± 0.0.41 mm was observed at a concentration of 0.625 mg/mL. These results are promising and suggest that LW-XG can be used as an antibacterial material in dentistry. For this purpose, the antibacterial activity of LW-XG also needs to be tested against other bacteria that can cause peri-implantitis. The antibacterial activity of LW-XG against *S. Aureus* may be due to its low molecular weight and hydroxyl groups. It is known that antibacterial polysaccharides can disrupt the cell wall and the membrane of bacteria or destroy the entire cell protein and cell membrane protein [78,90]. According to Wang et al., LW-XG increased the cell membrane permeability of *S. aureus* but had almost no effect on the cell wall. LW-XG could inhibit biofilm formation. Ca^2+^, Mg^2+^-ATPase is a calcium pump on the cell membrane that can hydrolyze ATP to pump intracellular Ca ions to the extracellular membrane, thus maintaining a relatively low intracellular Ca ion concentration. This maintains cell stability and ensures normal cell function. Ca ions are an important regulator of various cellular processes in all cells and a central factor in cytoplasmic function. The accumulation of Ca ions in the cytoplasm leads to an increase in reactive oxygen species, resulting in cytoplasmic dysfunction and cell apoptosis [165,166,167]. The effect of LW-XG against *S. aureus* showed that LW-XG not only affected cell membrane permeability but also inhibited biofilm formation and Ca^2+^-Mg^2+^-ATPase activity on the cytomembrane of *S. aureus* [139].

It would also be important to investigate the bioactivity of LW-XG for its application in implantology.

## 4. Perspectives for the Application of Antibacterial Polysaccharides in Dental Implantology

Bacterial resistance to antibiotics forces researchers to find new antibacterial materials that can destroy bacteria without being detected by bacteria or causing bacterial mutations that lead to new resistance.

Polysaccharides are the most abundant biopolymers in nature and are produced by animals, plants, and microorganisms. Their abundance, bioactivity, structural diversity, low toxicity, and low side effects make them an attractive source for the development of new antibacterial materials for use in dental implantology. The challenge for research is to utilize natural sources to produce such materials for the benefit of patients. The functionalization of biopolymers to improve their antibacterial activity, biocompatibility, bioactivity, stability, and mechanical properties, in order to make them a sought-after material in dental implantology, is the current task of bone tissue engineering. Another area of research is the blending of antibacterial polysaccharides with other natural biomaterials, such as antibacterial peptides (AMPs), which are known for their potent antibacterial activity. It is important to ensure that the combination of both materials does not lead to a loss of antibacterial activity or an alteration of their toxicity.

## 5. Conclusions

As bacterial resistance to antibiotics increases, antibacterial polysaccharides are becoming promising materials for combating pathogenic bacteria in dental implantology because such bacteria pose a significant risk to the patient’s health. Polysaccharides (PSs) are the most readily available natural polymers with diverse physical properties, making them promising candidates in many biomedical fields. Polysaccharides have several advantages over other synthetic polymers, including being safe, economical, chemically stable, biocompatible, and biodegradable. Furthermore, they can be chemically tailored for specific application purposes. Antimicrobial polysaccharides have attracted considerable interest as potential antimicrobial agents because they act as the first line of defense against several pathogens. Several polysaccharides, such as chitosan and its derivatives, alginate, pectin, xanthan, etc., have been reported to reduce the growth of pathogens and their biofilms on implant surfaces. Compared to other antimicrobial coatings, polysaccharide-functionalized coatings offer several advantages, such as a broad application, rapid antibacterial action, and low toxicity. This makes them ideal therapeutic agents for implant coatings. The antibacterial activity of polysaccharides depends on their solubility, configuration, molecular size, molecular weight, surface area, monomers, concentration, functional groups, viscosity, and shape. Coatings containing antibacterial polysaccharides can protect dental implants because of their contact and release bactericidal activities and osteogenic activities. In recent years, the use of polysaccharides in bactericidal coatings for dental implants has been intensively researched. These coatings are expected to play an important role in the prevention and treatment of dental implant-associated infections.

## Figures and Tables

**Table 1:** A summary of antibacterial polysaccharides that can be used in dental implantology.

Material	Properties	Modification	Antibacterial Activity	Mechanism	Ref.
Chitosan	high antibacterial activity, biocompatibility, biodegradability, nontoxicity, hemostatic effect, rigidity, and brittleness	no	*S. aureus* *E. coli* *S. gordonii* *S. epidermis* *Agg. actinomycetemcomitans* *P. gingivalis*	Electrostatic interaction between chitosan and bacteria.	[100,104,114,117,130]
Chitosan	biocompatibility with a positive effect on fibroblast proliferation, hemostatic properties, anti-inflammatory effect, antibacterial activity, anticancer, antitumor	carboxymethyl chitosan *(O-CMS)* *(N-CMS)* *(N,O-CMS)*	*S. aureus* *E. coli* *P. aeruginosa*	Electrostatic interaction between carboxymethyl chitosan.	[131,132,133,134,135]
Chitosan	biocompatibility, soluble in physiologic pH, antibacterial	quaternary ammonium trimethyl chitosan (QCMC)	*E, Coli* *P. aeruginosa* *S. Aureus* *E. faecalis*	Electrostatic interaction between positively charged ammonium groups with negatively charged bacteria.	[131,136,137]
Pectin (low-methoxyl commercial citrus pectin)	gelling, thickening, biocompatibility, biodegradability, anticancer, antibacterial, corrosion inhibitor, used to load and control drug release	no	*Planktonic bacteria* at low pH *E. coli* *S. pyogenes* *S. aureus* *E. faecalis*	The mechanisms of antibacterial activity are still not understood. The complexity of the structure, differences in extraction methods, and different fragmentation techniques make it hard to determine the active groups.	[126,127]
Lemon IntegroPectin	high content of polyphenols, flavonols such as kaempferol, phenolic acids (*p*-coumaric acid and gallic acid), and monoterpenoid saffron	no	*E. coli* *P. aeruginosa*	Synergistic mechanisms involving the intrinsic antibacterial activity of the pectic polymer and antibacterial activity of citrus flavonoids and terpenes at the surface of IntegroPectin.	[128]
Alginate (brown seaweeds)	film-forming ability, gelling, nontoxicity, pH responsiveness, hydrophilicity, biocompatibility, biodegradability, wound healing, carriers for drug delivery	mix with AgNPs	*S. aureus* *E. coli* *S. Epidermis*	Ag+ can bind the thiol group of bacteria proteins and interfere with DNA replication.	[138]
Xanthan oligosaccharide	lowering of the transcriptional levels of genes (fnbA, fnbB, and dfB) related to biofilm formation	biodegradation of commercial xanthan	*S. aureus*	Increase in cell membrane permeability.	[139]

## Data Availability

Not applicable.
